# Advances in Carbon Dot-Based Optical (Bio)Sensors for Contaminant Detection in Wastewater-Based Epidemiology

**DOI:** 10.3390/s26082362

**Published:** 2026-04-11

**Authors:** Ricarda Torre, Luís Pinto da Silva

**Affiliations:** Chemistry Research Unit (CIQUP), Institute of Molecular Sciences (IMS), Department of Geosciences, Environment and Spatial Plannings, Faculty of Sciences, University of Porto, R. Campo Alegre s/n, 4169-007 Porto, Portugal; ricarda.torre@fc.up.pt

**Keywords:** wastewater-based epidemiology, optical biosensors, fluorescent carbon dots, green synthesis, waste-derived nanomaterials, wastewater monitoring, public health surveillance

## Abstract

Wastewater-based epidemiology (WBE) has emerged as a powerful approach for population-level monitoring of chemical exposure, health status, and disease transmission by analysing wastewater. Although chromatographic and molecular techniques remain the gold standard in WBE, their high cost, infrastructural demands, and limited suitability for decentralized and real-time monitoring motivate the development of complementary sensing technologies. In this context, optical (bio)sensors, particularly fluorescence-based platforms, have attracted increasing attention due to their high sensitivity, rapid response, and potential for on-site monitoring. This review discusses recent advances in fluorescent optical (bio)sensors for WBE, with a particular focus on carbon dots (CDs), including waste- and biomass-derived CDs produced via green synthesis as well as CDs obtained from commercial chemicals. The applicability of CD-based sensors to wastewater-relevant analytes is evaluated, highlighting current achievements, as well as existing limitations and challenges related to real-sample validation and the translation of these platforms into robust, field-deployable systems for their implementation in sustainable wastewater monitoring and public health surveillance.

## 1. Wastewater-Based Epidemiology

Wastewater-Based Epidemiology (WBE) is a population-level surveillance approach based on the analysis of chemical and biological biomarkers present in wastewater to infer patterns of exposure, consumption, health status, and disease circulation within a community [1,2]. The underlying principle of WBE is that substances consumed by humans are excreted in urine and feces, either unchanged or as metabolites, and subsequently conveyed through sewage systems to wastewater treatment plants (WWTPs). By sampling and analysing wastewater before treatment, integrated information representative of both symptomatic and asymptomatic individuals can be obtained, while maintaining low operational costs and safeguarding individual privacy [3].

Originally, WBE was developed for monitoring enteric pathogens, such as poliovirus, norovirus, rotavirus, adenovirus, and other enteroviruses, enabling assessment of viral circulation and early detection of outbreaks in defined geographic areas [4,5]. Since the 1990s, its application has progressively expanded beyond pathogen surveillance to include the estimation of community-wide consumption of illicit drugs, marking a pivotal transition toward broader public health and environmental monitoring [6,7]. Over the past two decades, WBE has evolved into a versatile and non-invasive tool for assessing population-level exposure to a wide range of chemical stressors. Its scope now encompasses the evaluation of illicit and licit drug use, exposure to heavy metals and environmental pollutants, the spread of infectious diseases, and the prevalence of antibiotic resistance genes (ARGs) [8,9]. More recently, the global application of WBE to monitor SARS-CoV-2 has further demonstrated its value as an effective early-warning and decision-support system during public health emergencies [10,11,12]. The successful implementation of WBE relies on the identification of robust, representative (bio)markers that accurately reflect human behaviour, health status, and environmental exposure.

Nevertheless, the heterogeneous and dynamic nature of wastewater presents substantial analytical challenges, as its complex matrix contains a wide range of interfering substances. This complexity emphasizes the necessity for sensitive, selective, and rigorously validated analytical methodologies to ensure reliable biomarker identification and quantification.

A variety of fluorescent nanomaterials have been explored for optical sensing applications, including semiconductor quantum dots, graphene-based materials, and organic fluorophores. Semiconductor quantum dots exhibit high fluorescence quantum yields and excellent photostability; however, their potential toxicity and environmental concerns, often associated with heavy metal content, limit their applicability in sustainable sensing platforms. Graphene oxide and related carbon-based materials offer strong quenching capabilities and large surface area, but their fluorescence properties are generally less tunable and often require complex functionalization. Organic fluorophores, while widely used, may suffer from photobleaching and limited stability in complex matrices [13,14].

In this context, carbon dots (CDs) emerge as particularly attractive alternatives, combining low toxicity, environmental friendliness, cost-effective synthesis—especially from waste-derived precursors—and tunable optical properties [15].

Therefore, the development of CD-based fluorescent (bio)sensors represents a promising strategy to address current analytical limitations in WBE. CDs can be synthesized from a wide range of precursors, including waste-derived materials, enabling more sustainable and cost-effective production routes aligned with green chemistry principles. In addition to their favorable optical properties, carbon dot-based (bio)sensors offer several practical advantages, including low sample volume requirements, rapid response, high sensitivity and selectivity, and the potential for low-cost, portable device integration. These characteristics make them particularly attractive for decentralized and real-time monitoring applications. This review aims to provide a comprehensive overview of recent advances in CD-based optical sensing platforms for the detection of contaminants and biomarkers in the context of wastewater-based epidemiology, while also considering their applicability in other complex matrices.

### 1.1. Global Use of WBE in Real-Time

The real-time application of WBE has become an increasingly important component of global public health surveillance, enabling near-continuous monitoring of disease circulation and other population-level health indicators. By capturing temporal trends in pathogen loads and community exposure ahead of clinical reporting, real-time WBE provides early-warning signals that complement conventional epidemiological surveillance systems, which are often affected by testing delays, underreporting, and healthcare-seeking behaviour.

Consequently, wastewater surveillance has been recognized as a valuable decision-support tool for timely public health interventions and preparedness planning [16,17]. A flagship example of real-time WBE implementation is the response to the COVID-19 pandemic. National and international public health institutions rapidly established wastewater surveillance networks that provided weekly (and in some cases daily) updates on SARS-CoV-2 levels in sewage. These datasets were often publicly accessible through dashboards, facilitating transparent communication of viral trends to both health authorities and the general public. In the United States, the Centers for Disease Control and Prevention (CDC) incorporated wastewater data into its core public health data systems, showing weekly updates on multiple respiratory pathogens (e.g., SARS-CoV-2, influenza A, and RSV) to support early warning and informed health decision-making by communities and policymakers.

This real-time data submission goal—where a large proportion of samples are reported within seven days of collection—reflects efforts to accelerate detection of public health threats and optimize resource allocation [18]. At the global level, the World Health Organization (WHO) maintains a COVID-19 wastewater surveillance dashboard that aggregates publicly available national and regional datasets, highlighting the widespread adoption of environmental surveillance for SARS-CoV-2 across all WHO regions.

**Figure 1:**
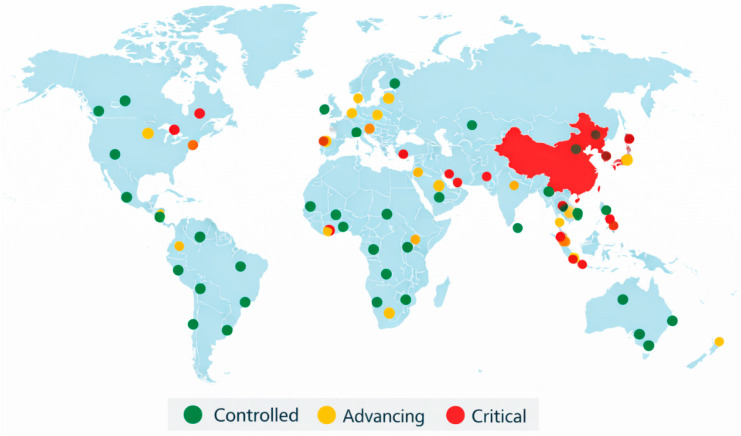
Conceptual illustration of a hypothetical global wastewater-based epidemiology surveillance framework. Color-coded indicators represent illustrative epidemiological trends inferred from wastewater monitoring (green: controlled; yellow: advancing; red: critical). This figure is intended for conceptual demonstration purposes only and does not represent real epidemiological data. The illustration was created by the authors based on existing wastewater surveillance platforms, including the JRC Wastewater Observatory [21].

The WHO underscores that wastewater data provide valuable early signals of changes in virus circulation—often preceding increases in reported clinical cases and hospital admissions by several weeks—and should be used alongside traditional indicators such as case counts and hospitalization data. Member States are encouraged to strengthen and maintain SARS-CoV-2 wastewater monitoring and to make these data publicly available to support collective efforts in outbreak detection, variant tracking, and public communication [19,20]. Figure 1 presents a conceptual illustration of how wastewater-based epidemiology can support real-time epidemiological surveillance at a global scale. This schematic does not represent actual epidemiological data but rather a hypothetical scenario designed to highlight the potential integration of wastewater monitoring into public health decision-making frameworks. The illustration was developed by authors based on existing wastewater surveillance initiatives and visualization approaches, including platforms such as European Commission’s Joint Research Centre (JRC) Wastewater Observatory [21].

### 1.2. Major Contaminants Tracked Through WBE

Wastewater-based epidemiology has been applied to monitor a wide range of contaminants that reflect population-level exposure, behaviour, and health status. Among the primary targets of WBE are infectious agents, illicit and recreational drugs, pharmaceuticals and personal care products, environmental xenobiotics, and microbiological determinants such as antibiotic resistance [22,23].

One of the most widely studied contaminant classes in WBE is viral pathogens, particularly SARS-CoV-2, whose RNA has been quantified in municipal wastewater to infer community infection dynamics and provide early warning of outbreaks. Numerous studies have demonstrated strong correlations between viral RNA loads in wastewater and clinical COVID-19 cases, with signals often preceding reported incidence data by several days, highlighting WBE’s utility in infectious disease surveillance at the population scale [24,25].

WBE has also been extensively used to track illicit and recreational drug use [26]. Quantification of human metabolites of substances such as cocaine [27,28], methamphetamine [29,30], MDMA [31,32], and opioids [33,34] in sewage enables objective estimation of drug consumption trends across communities and over time. Such studies have been conducted globally, providing spatial and temporal patterns of drug use that complement traditional public health and law enforcement datasets. For example, the mass loads of these metabolites in wastewater can be used to calculate per capita consumption rates when combined with biomarker excretion rates and flow data, facilitating direct comparisons between cities and regions.

Another major application of WBE concerns the assessment of pharmaceuticals and personal care products (PPCPs), which enter wastewater systems primarily through human excretion following consumption, as well as through improper disposal [26]. Numerous studies have demonstrated the applicability of WBE to estimate community-level consumption of specific pharmaceutical classes, including antidepressants such as fluoxetine [35], sertraline [36], citalopram [37], and venlafaxine [38]; non-steroidal anti-inflammatory drugs (NSAIDs) such as ibuprofen [39], diclofenac [40], naproxen [41], and ketoprofen [42]; and antibiotics including amoxicillin [43], ciprofloxacin [44], sulfamethoxazole [45], and azithromycin [46]. In addition, widely consumed psychoactive and lifestyle-related substances, such as caffeine [47], nicotine (via cotinine) [48], and paracetamol [49], are frequently monitored as robust population biomarkers. Wastewater-based studies have revealed pronounced temporal variations in the consumption of these compounds, reflecting seasonal disease prevalence, public health interventions, and behavioural changes during the COVID-19 pandemic. Furthermore, comparisons between pharmaceutical loads measured in wastewater and external data sources—such as prescription records, sales statistics, and healthcare utilization data—have demonstrated good agreement in many cases, supporting the validity of WBE as a complementary tool for evaluating drug consumption patterns and broader public health trends. Beyond intentional exposure, WBE provides insights into unintentional human exposure to environmental contaminants.

Comprehensive reviews have documented the detection of organic chemicals such as organophosphorus flame retardants, per- and polyfluoroalkyl substances (PFAS), phthalates and terephthalates, benzotriazoles and benzothiazoles, bisphenols, pesticides, and parabens in wastewater, reflecting broad exposure to these potentially harmful compounds at the population level [23,26,50]. For instance, WBE studies have reported the presence of multiple PFAS compounds in influent wastewater from urban treatment plants, demonstrating the technique’s applicability to persistent and bioaccumulative contaminants [51,52,53].

In addition to organic micropollutants and pharmaceuticals, WBE has also been applied to assess population-level exposure to inorganic contaminants, particularly trace metals. Metals such as lead, mercury, cadmium, copper, and iron can enter wastewater systems through industrial discharges, corrosion of water distribution systems, and domestic activities, and their presence in sewage has been proposed as an indicator of environmental and occupational exposure at the community level. Several studies have demonstrated the feasibility of monitoring metal loads in influent wastewater to identify spatial and temporal trends in exposure, supporting the inclusion of metal ions among relevant WBE targets [3,54,55].

Pesticides constitute another important class of contaminants addressed within the WBE framework. Agricultural runoff, urban pest control, and food consumption contribute to the occurrence of pesticide residues and their metabolites in wastewater. Compounds such as neonicotinoids [56,57], organophosphates [58,59], triazoles [60,61], and organochlorine pesticides [62,63] have been detected in wastewater, reflecting both dietary intake and environmental exposure. Wastewater-based monitoring of these substances provides valuable information on regional agricultural practices, seasonal application patterns, and potential human health risks associated with chronic low-level exposure.

Naturally occurring bioactive compounds, including flavonoids and other polyphenols, have emerged as additional targets of interest in residual water analysis [64,65,66]. Although not contaminants per se, flavonoids originate from plant-based foods, beverages, and herbal products and can be excreted by humans or released during food processing and industrial activities. Their detection in wastewater may serve as indirect markers of dietary habits, food consumption patterns, or specific industrial discharges. Collectively, the inclusion of metals, pesticides, and flavonoids further highlights the broad scope of wastewater-based epidemiology as a tool capable of capturing both harmful exposures and lifestyle-related biomarkers at the population level.

**Figure 2:**
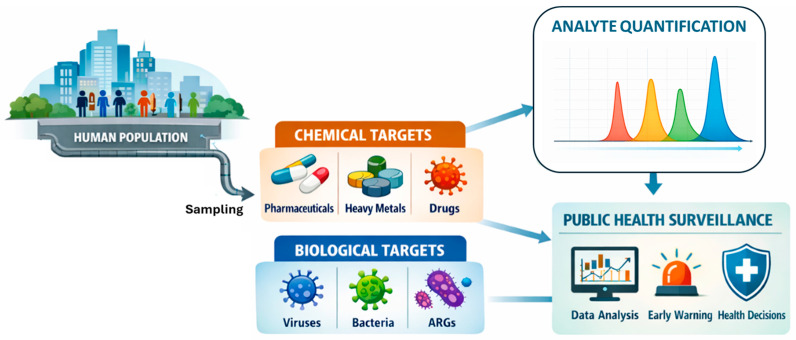
Schematic representation of wastewater-based epidemiology workflow. Biomarkers originating from the human population are collected through sampling and classified into chemical and biological targets. Quantification of target analytes is achieved through analytical signal responses, enabling data-driven public health surveillance, including early warning and decision-making processes.

Finally, WBE is increasingly applied to monitor antibiotic resistance genes (ARGs) [67] and other microbial determinants associated with antimicrobial resistance (AMR) [68]. Wastewater serves as a reservoir where resistant bacteria and ARGs from human sources converge, providing a population-scale indicator of resistance dissemination. Recent work highlights the detection of clinically relevant ARGs in sewage, underscoring the importance of WBE for public health monitoring and the evaluation of interventions aimed at reducing AMR spread [69]. The overall workflow of wastewater-based epidemiology is illustrated in Figure 2. The process begins with the human population as the primary source of chemical and biological markers released into wastewater. Following sampling, these biomarkers are analyzed to identify relevant chemical targets (e.g., pharmaceuticals, heavy metals, and drugs) and biological targets (e.g., viruses, bacteria, and antibiotic resistance genes). Quantitative analysis of the target analytes is then performed based on analytical signal responses, enabling accurate determination of concentration levels. The resulting data are subsequently integrated into public health surveillance systems, supporting data analysis, early warning strategies, and informed decision-making.

### 1.3. Common Analytical Methods for Contaminant Detection in WBE

Analytical strategies in wastewater-based epidemiology are largely centered on liquid chromatography–mass spectrometry (LC–MS), as most biomarkers of interest consist of polar human metabolites rather than their parent compounds [70].

In parallel, polymerase chain reaction (PCR)-based methods are routinely employed for the detection of viral nucleic acids in wastewater, including poliovirus, hepatitis B virus, and SARS-CoV-2, although such approaches can be susceptible to matrix inhibition and require careful optimization of concentration and recovery steps [71]. Although highly effective, PCR-based approaches require specialized laboratory infrastructure, skilled personnel, and carefully optimized sample concentration and recovery procedures. In contrast, liquid chromatography–tandem mass spectrometry (LC–MS/MS) offers several advantages, such as shorter analytical run times, high sensitivity, and the ability to simultaneously detect viral proteins and host-derived biomarkers through both targeted and untargeted workflows. Mass spectrometry has been highlighted as a powerful tool to characterize a broad range of WBE targets by generating a comprehensive chemical and proteomic fingerprint of wastewater samples [72]. This comprehensive detection capability enables earlier and more robust surveillance. Notably, LC–MS/MS-based studies have reported the identification of SARS-CoV-2-associated proteins in wastewater up to six days before corresponding clinical case data were available, including peptides derived from structural and accessory proteins, an observation that underscores the potential of proteomic biomarkers such as membrane, spike, and nucleocapsid proteins for COVID-19 monitoring [71,73]. Despite the high analytical performance of these conventional techniques, their application is often limited in scenarios requiring rapid, decentralized, and continuous monitoring. In this context, optical sensing strategies, particularly those based on carbon dots, offer distinct advantages for on-site, real-time analysis, where low-cost instrumentation, minimal sample preparation, and rapid response are essential. These features make them especially suitable for distributed monitoring networks and early warning systems within wastewater-based epidemiology. Despite the high analytical performance of these conventional techniques, their application is often limited in scenarios requiring rapid, decentralized, and continuous monitoring. In this context, optical sensing strategies, particularly those based on CDs can offer distinct advantages for on-site and real-time analysis, where low-cost instrumentation, minimal sample preparation, and fast response are essential. These features make them especially suitable for distributed monitoring networks and early warning systems within wastewater-based epidemiology [74].

## 2. Emerging Optical (Bio)sensor Technologies for Wastewater Monitoring

Optical (bio)sensors are analytical devices that transduce chemical or biological recognition events into measurable optical signals, such as changes in absorbance, fluorescence intensity, emission wavelength, or luminescence lifetime.

These platforms are particularly attractive for wastewater monitoring due to their high sensitivity, fast response times, and suitability for non-invasive analysis in complex matrices, including environmental and biological samples [75,76,77]. Among the different optical detection strategies, fluorescence-based sensing is one of the most widely explored approaches. Fluorescence sensing relies on the excitation of a fluorophore, followed by emission at a longer wavelength, with variations in emission intensity or spectral properties correlating with the presence and concentration of target analytes. This strategy has been extensively applied to the detection of wastewater-relevant species, including metal ions, pharmaceuticals, pesticides, pathogens, and biomarkers related to human health and environmental exposure [77,78,79].

Carbon dots (CDs) have emerged as an important class of fluorescent nanomaterials employed in optical (bio)sensors. CDs are nanoscale carbon-based materials, typically below 10 nm in size, characterized by strong and tunable photoluminescence, good water solubility, high photostability, and low toxicity. Their fluorescence emission is generally attributed to a combination of core and surface states. These properties make CDs particularly suitable for sensing applications in aqueous and chemically complex environments such as wastewater [76,79,80].

In addition to CDs, optical sensing platforms frequently incorporate other functional materials to improve performance. Graphene oxide and related carbon materials are commonly used as fluorescence quenchers or energy acceptors due to their broad absorption profiles and strong π–π interactions. Polymers, silica-based matrices, and metal-containing components are also employed to enhance stability, selectivity, and signal transduction, particularly in hybrid sensing architectures designed for complex environmental samples [81,82,83]. Fluorescence-based optical sensing using CDs typically operates via signal modulation mechanisms, such as fluorescence quenching or enhancement. In “turn-off” sensors, analyte interaction induces fluorescence quenching via mechanisms such as static or dynamic quenching, or inner-filter effects. Conversely, “turn-on” sensors rely on fluorescence recovery or enhancement upon analyte binding, often providing improved signal-to-noise ratios. Ratiometric and FRET-based strategies are also frequently reported to improve analytical reliability in complex matrices [77,84,85].

To ensure reliable fluorescence measurements and enable comparison between different sensing systems, reference fluorophores are commonly used. Classical molecular fluorophores such as quinine sulfate, fluorescein, rhodamine derivatives, and coumarins are widely reported as fluorescence standards for calibration and quantum yield determination in optical sensing studies, including those involving CDs [78,86]. The sensing performance of carbon-dot-based platforms is further enhanced by their ease of surface functionalization.

Abundant surface functional groups enable conjugation with biorecognition elements such as antibodies, aptamers, enzymes, and molecularly imprinted polymers, facilitating selective detection of biological targets. Moreover, coordination with metal ions or incorporation into hybrid nanocomposites can introduce additional signal amplification pathways, expanding the applicability of fluorescent optical biosensors for wastewater monitoring [79,81,83].

Overall, the combination of fluorescence-based optical transduction, carbon-dot nanomaterials, and advanced functionalization strategies positions optical (bio)sensors as promising tools for wastewater monitoring and wastewater-based epidemiology. Their sensitivity, adaptability, and compatibility with complex matrices support their application in environmental surveillance, public health monitoring, and emerging decentralized analytical platforms, although challenges related to matrix effects, reproducibility, and long-term stability remain [76,77,86].

### Application of Waste-Derived Carbon Dots for Optical Detection

This section provides an overview of the fundamental properties, synthesis strategies, and functional characteristics of CDs, with particular emphasis on waste-derived materials and their relevance for the development of fluorescent (bio)sensing platforms.

Since their initial discovery during the purification of carbon nanotubes in 2004, CDs have emerged as promising alternatives to conventional semiconductor quantum dots due to their superior photostability, environmental friendliness, and ease of surface functionalization [87,88].

Structurally, CDs consist of an amorphous or partially graphitic carbon core containing sp^2^/sp^3^-hybridized domains, surrounded by abundant surface functional groups such as hydroxyl, carboxyl, and amino moieties, which play a crucial role in their optical behavior and sensing performance [89]. In recent years, increasing emphasis has been placed on developing waste-derived CDs as part of sustainable, circular-economy-driven nanomaterial strategies.

Waste-derived CDs are synthesized from biomass or waste resources, including food waste, agricultural residues, plant by-products, and industrial biomass, enabling the valorization of low-value materials into high-performance fluorescent nanomaterials [89,90]. Representative examples include CDs prepared from fruit peels, such as banana, orange, and apple waste; spent coffee grounds and tea waste; rice husk; corn silk; and other plant-derived residues rich in carbohydrates, proteins, and polyphenols [88,91,92]. Beyond their availability as carbon sources, the chemical composition of these waste precursors plays a decisive role in determining the physicochemical structure, surface chemistry, and optical performance of CDs, establishing a clear precursor–property–performance relationship. Biomass-derived precursors are inherently composed of varying proportions of carbohydrates, proteins, lipids, and polyphenolic compounds, which directly influence the carbonization process, heteroatom incorporation, and surface functionality of the resulting CDs [93]. In general, carbohydrate-rich precursors (e.g., fruit peels or lignocellulosic residues) favour the formation of more carbonized and graphitic cores, typically leading to stable fluorescence emission but relatively limited surface passivation. In contrast, nitrogen-rich precursors such as coffee grounds, tea waste, or protein-containing biomass promote intrinsic N-doping during synthesis, significantly enhancing quantum yield, electron transfer capability, and sensing sensitivity [88,94]. For instance, CDs derived from spent coffee grounds exhibit abundant nitrogen- and oxygen-containing functional groups (e.g., hydroxyl, carboxyl, and amino groups), originating from the complex composition of cellulose, hemicellulose, and proteins in the precursor. These surface functionalities improve water dispersibility, fluorescence stability, and provide active sites for selective interactions with analytes, enabling highly sensitive and selective detection in environmental and food matrices [94]. Similarly, precursors rich in polyphenols or organic acids contribute to oxygen-functionalized surfaces, which enhance solubility and facilitate coordination with metal ions or biomolecules, thereby improving selectivity in sensing applications. In addition, heteroatom co-doping (e.g., N, S) derived from natural precursors has been shown to modulate the electronic structure of CDs, leading to tunable emission behavior, higher quantum yields, and improved analytical performance [95]. Therefore, the rational selection of waste precursors enables the controlled tuning of CD properties without requiring extensive post-synthetic modification. This composition-driven approach is particularly relevant for the design of fluorescent (bio)sensors, where surface chemistry, heteroatom doping, and photoluminescence properties directly govern sensitivity, selectivity, and detection limits [93].

The synthesis of waste-derived CDs aligns closely with green synthesis principles, aiming to minimize hazardous reagents, reduce energy consumption, and avoid complex purification steps. Compared to conventional chemically derived CDs, green CDs synthesized from renewable resources are generally cost-effective, environmentally benign, and suitable for applications requiring low toxicity, such as biosensing and bioimaging [90,96]. Importantly, the choice of precursor and synthesis conditions has a significant influence on the optical properties, quantum yield, and surface chemistry of the resulting CDs, which directly affects their sensing performance [88,92].

From a synthetic perspective, CDs can be produced via both top-down and bottom-up approaches. Top-down methods involve the breakdown of bulk carbonaceous materials into nanoscale CDs using techniques such as laser ablation, arc discharge, electrochemical oxidation, or chemical oxidation. Although effective, these methods often require harsh conditions, sophisticated instrumentation, and offer limited control over particle size and surface chemistry, making them less compatible with green synthesis strategies [90,91]. Consequently, bottom-up approaches are more widely adopted, particularly for waste-derived and green CDs. These methods rely on the carbonization of small organic molecules or biomass precursors through processes such as hydrothermal or solvothermal treatment, microwave-assisted synthesis, ultrasonic treatment, and pyrolysis [91,92].

Among bottom-up strategies, hydrothermal synthesis is the most frequently reported method for waste-derived CDs, owing to its simplicity, scalability, moderate reaction conditions, and compatibility with aqueous biomass precursors. This approach enables effective carbonization and surface passivation in a single step, often yielding CDs with high fluorescence intensity and tunable emission properties [90,97]. Microwave-assisted synthesis has also gained attention due to its rapid reaction times, uniform heating, and reduced energy consumption, making it particularly attractive for green and large-scale production of CDs from food waste and plant residues [91,92].

**Figure 3:**
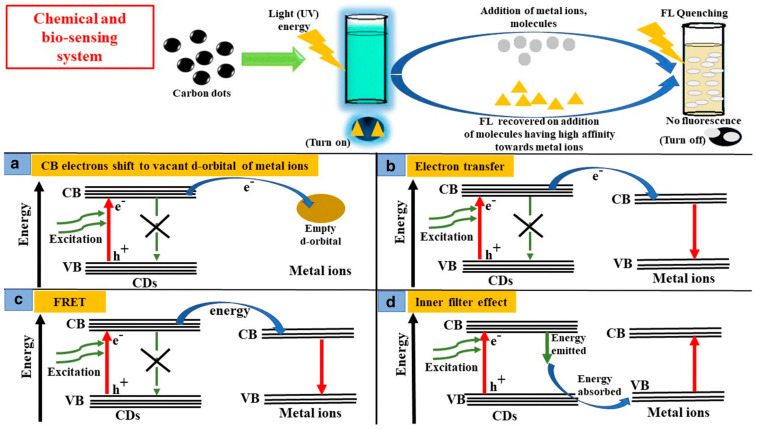
Role of carbon dots (CDs) in chemical and biosensing based on fluorescence quenching mechanisms. Changes in fluorescence intensity may occur through different pathways, including (**a**) transfer of conduction band electrons from CDs to low-lying vacant d-orbitals of metal ions, (**b**) electron transfer between the conduction bands of CDs and metal ions, (**c**) fluorescence resonance energy transfer (FRET), and (**d**) inner filter effect. Reproduced from [100] under a Creative Commons Attribution 4.0 International License.

The unique fluorescence properties of CDs underpin their widespread application in optical sensors and biosensors. CD-based fluorescent sensing typically relies on mechanisms such as fluorescence quenching or enhancement, inner filter effects, and Förster resonance energy transfer (FRET), which can be modulated by interactions between the CD surface and target analytes (Figure 3) [87,98]. The presence of abundant surface functional groups facilitates selective interactions with metal ions, organic pollutants, biomolecules, and pathogens, enabling sensitive and selective detection. Furthermore, the ease of surface functionalization allows the integration of CDs with biorecognition elements such as antibodies, aptamers, and enzymes, expanding their applicability to fluorescent biosensing platforms [98,99].

Overall, the combination of waste-derived precursors, green synthesis routes, and favorable optical properties positions CDs as highly attractive materials for next-generation fluorescent (bio)sensors. These characteristics are particularly relevant for applications in wastewater-based epidemiology, where robust, sensitive, and cost-effective detection strategies are required for complex matrices. Despite these advantages, challenges related to reproducibility, quantum yield optimization, and matrix effects remain key aspects to be addressed in future developments [89,97].

## 3. Fluorescent Carbon-Dot-Based (Bio)sensors Targeting Wastewater-Relevant Analytes

This section provides a structured discussion of the studies summarized in Table 1, focusing on fluorescent carbon dot-based (bio)sensors for the detection of analytes relevant to wastewater-based epidemiology. The analysis is organized according to target categories and key analytical parameters, including synthesis strategy, quantum yield, detection limits, and performance in real samples.

Table 1 compiles research articles that employ fluorescent CDs in sensing and biosensing platforms for the detection of a wide range of chemical and biological targets relevant to human health and wastewater-based epidemiology. Given the limited number of studies directly validated in wastewater matrices, the scope of this analysis was expanded to include sensors evaluated in other complex matrices, provided the targeted analytes are known to occur in wastewater and pose potential public health risks. This approach reflects the current state of the art and enables the identification of transferable sensing strategies relevant to WBE applications. With respect to the target and recognition scheme, a clear predominance of sensors targeting small-molecular analytes, particularly metal ions, inorganic anions, and antibiotics, is observed.

Metal ions such as Fe^3+^, Cu^2+^, Pb^2+^, and Hg^2+^ are frequently detected through fluorescence quenching or enhancement mechanisms based on direct interactions between the analyte and functional groups on the CD surface, often without the need for a recognition element [101,102,103,104,105,106]. These systems benefit from relatively simple architectures and good robustness, which is advantageous for application in complex matrices. Inorganic anions, including fluoride and cyanide, are also addressed using similar fluorescence-based strategies, demonstrating the versatility of CDs for non-metal ionic species of environmental relevance [104,107].

CD-based sensors developed for metal ion detection display substantial variability in both precursor selection and optical performance. Reported carbon sources include waste-derived materials such as banana peels [101,102], orange peels [105], and rice husk [106], as well as synthetic precursors including citric acid and succinic acid [103]. The resulting quantum yields span a wide range, from as low as 2.6% [101] to as high as 56.2% [106], corresponding to nearly a 20-fold difference. Despite this pronounced variability, all reported sensors achieved micromolar to sub-micromolar limits of detection for environmentally relevant metal ions, including Fe^3+^, Cu^2+^, Pb^2+^, and Hg^2+^. Notably, the lowest detection limit reported was 0.01335 µM for Pb^2+^ [104]. Importantly, high quantum yield did not consistently translate into improved analytical sensitivity. For example, banana peel-derived CDs with relatively modest quantum yields (2.6–18%) [101] exhibited detection limits comparable to those achieved using rice husk-derived CDs with significantly higher quantum yield (56.2%) [106]. This observation suggests that surface chemistry and the availability of functional groups involved in metal coordination may play a more decisive role than intrinsic fluorescence brightness in determining sensing performance. Recovery studies generally demonstrated good analytical accuracy, with values ranging from 89% to 116% in tap, river, and irrigation water samples. However, only a single study [104] evaluated sensor performance in real wastewater, highlighting the need for further validation under realistic WBE conditions.

With respect to antibiotics, they represent one of the most extensively investigated target classes, reflecting their widespread use in human and veterinary medicine and their frequent detection in wastewater. CD-based sensors developed for antibiotic detection also display considerable variability in precursor selection, synthesis routes, and optical properties. Reported carbon sources include waste-derived precursors such as rice husk [106,108], sumac [109], and spent coffee grounds [94], as well as synthetic molecular precursors including citric acid [110,111], sucrose [112,113], phosphoric acid [112], and formamide [114]. As a result, the reported quantum yields span a wide range, from as low as 1.4% for sumac-derived CDs [109] to values as high as 56.2% and 55.2% for rice husk-derived systems [106,108]. Despite this pronounced variability in fluorescence efficiency, all sensors achieved detection limits within environmentally relevant ranges for antibiotics such as ciprofloxacin, ofloxacin, tetracycline, oxytetracycline, kanamycin, and amoxicillin. Reported limits of detection range from the micromolar level down to the low nanomolar and subnanomolar levels, with the lowest values reaching 0.0007 µM for ofloxacin [109] and 0.00173 µM for tetracycline in swine wastewater [112]. As observed for metal ion sensing, high quantum yield does not consistently correlate with improved analytical sensitivity. For instance, sumac-derived CDs with a very low quantum yield (1.4%) [109] achieved detection limits comparable to or lower than those obtained using rice husk-derived systems with quantum yields exceeding 50% [106,108], indicating that surface functionalization and specific interactions between antibiotic molecules and CD surface groups play a more decisive role than intrinsic fluorescence brightness. Recovery experiments generally demonstrated good analytical accuracy across a wide range of sample matrices, with most reported values falling between approximately 89% and 110%. Satisfactory recoveries were obtained in relatively simple matrices such as tap, river, and lake water, as well as in more complex samples including milk, liquid eye drops, tablets, supplements, and swine wastewater [106,109,110,112,114]. These results indicate that the majority of antibiotic sensors maintain acceptable quantitative performance even in matrices with varying levels of organic and inorganic complexity.

Fluoride (F^−^) and cyanide (CN^−^) are also represented in Table 1 as relevant inorganic targets with recognized environmental and public health significance. CD-based fluorescent sensors reported for fluoride detection were synthesized using molecular precursors such as citric acid and glutamine, with sodium sulphide employed as a surface-modifying agent, yielding quantum yields of approximately 10% [104]. Despite this moderate optical efficiency, the sensor achieved a sub-micromolar limit of detection of 0.04317 µM within a linear range of 0–12 µM. Performance validation was conducted in complex matrices, including fish extracts and wastewater, with recovery values ranging from 89.30% to 116.40% and 90.22% to 115.05%, respectively, indicating good analytical accuracy even in biologically and chemically complex samples [104].

Cyanide detection was achieved using CDs derived from corn silks via a hydrothermal synthesis route, resulting in a quantum yield of approximately 15% [107]. The reported sensor exhibited a detection limit of 0.459 µM over a linear range of 0.5–50 µM and was successfully applied to diverse matrices, including industrial effluent, sorghum leaves, and flaxseeds. Although recovery data were not reported for cyanide detection, the demonstrated applicability across industrial and plant-based samples highlights the versatility of CD-based fluorescent platforms for monitoring highly toxic inorganic anions.

Regarding bacterial detection, CD-based fluorescent (bio)sensors are less extensively investigated than for other target classes, reflecting the greater complexity of biological targets compared to small molecules or inorganic ions.

The reported studies focus primarily on *Escherichia coli* as a model organism, employing nucleic acid-based biorecognition elements such as aptamers and oligonucleotides to confer selectivity [115,116,117]. CDs synthesized from waste-derived precursors, including orange peel [118] and carrot juice [119], as well as from synthetic precursors such as citric acid [120], were prepared using microwave-assisted or hydrothermal methods. When reported, quantum yields ranged up to 16.2% [118], although optical efficiency was not systematically correlated with analytical performance. Detection limits varied substantially depending on the recognition strategy, ranging from tens of CFU mL^−1^ to sub-100 CFU mL^−1^. Notably, an oligonucleotide-based sensor achieved a detection limit as low as 0.00103 µM equivalent (corresponding to approximately 60 CFU mL^−1^) in food matrices such as chicken, meat, and cheese [120], highlighting the sensitivity attainable through sequence-specific recognition.

**Figure 4:**
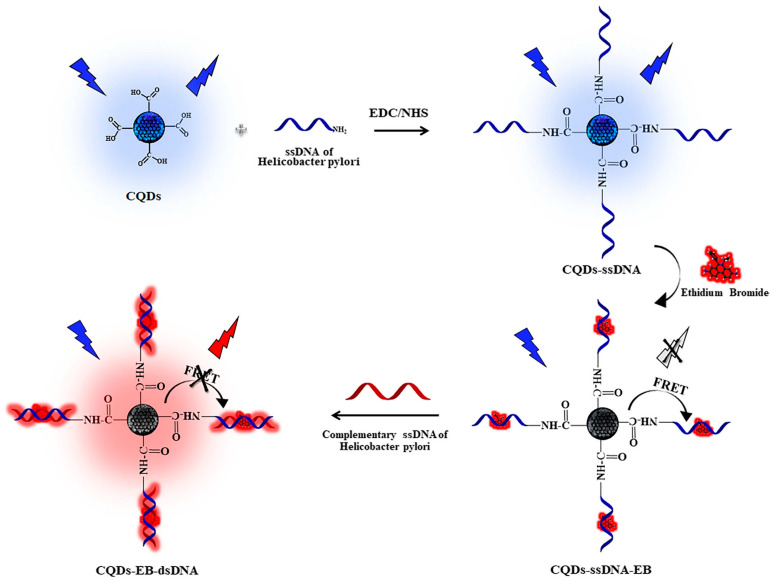
Schematic representation of a carbon dot (CD)-based fluorescence biosensor using a nucleic acid recognition strategy. Reproduced from [121] under a Creative Commons Attribution 4.0 International License.

Aptamer-based systems also demonstrated effective detection of *E. coli* in complex matrices, including milk [118], although recovery data were not consistently reported. To clarify the working principle of CD-based biosensors, a representative mechanism is illustrated in Figure 4. In this system, carbon dots are functionalized with single-stranded DNA (ssDNA) probes specific to the target sequence. In the absence of the target, the addition of ethidium bromide (EB) leads to fluorescence quenching, mainly due to fluorescence resonance energy transfer (FRET) between CDs (donor) and EB (acceptor), facilitated by spectral overlap. Upon introduction of the complementary DNA sequence, hybridization occurs, forming double-stranded DNA (dsDNA), which promotes the intercalation of EB into the DNA structure. This process alters the spatial arrangement and energy transfer efficiency within the system, resulting in fluorescence recovery or enhancement [118].

In addition to *E. coli*, a CD-based fluorescent biosensor targeting *Helicobacter pylori* was developed using ssDNA as the recognition element, achieving a detection limit of 0.098 µM with satisfactory recoveries (93.06–101.85%) in saliva samples [121]. Overall, these studies demonstrate that CD-based fluorescent biosensors can achieve sensitive and selective bacterial detection. However, their application remains largely limited to food and biological samples. Validation in wastewater matrices is notably absent, underscoring a significant gap that must be addressed before such platforms can be reliably integrated into wastewater-based epidemiology frameworks.

Viral detection using CD-based fluorescent (bio)sensors is primarily represented in Table 1 by platforms targeting SARS-CoV-2, reflecting the central role of wastewater surveillance during the COVID-19 pandemic. Both reported sensing strategies rely on antibody-based recognition elements [122,123], underscoring the suitability of immunoassays coupled with fluorescent CDs for viral biosensing. CDs were synthesized using thiourea and citric acid via a hydrothermal route [122] or obtained from a commercial source [123]. When reported, quantum yields reached values as high as 71.83% for commercially sourced materials [123]. These systems demonstrated sensitive detection in the nanogram per millilitre range, with limits of detection as low as 0.05 ng mL^−1^ and linear ranges from 0.1 to 100 ng mL^−1^. Performance validation was reported in serum samples, yielding recoveries between 110.05% and 115.45% [123]. Despite the promising analytical sensitivity achieved, validation of CD-based fluorescent biosensors for SARS-CoV-2 detection remains confined to biological matrices, with no systematic evaluation reported in real municipal wastewater, limiting their direct applicability to routine wastewater-based epidemiology.

Biological analytes including hormones, mycotoxins, and cytokine biomarkers represent an important subset of targets in Table 1, highlighting the applicability of fluorescent CD-based (bio)sensors to clinically and toxicologically relevant compounds.

Hormonal detection is exemplified by progesterone, monitored using an antibody-CD sensor synthesized from citric acid via a hydrothermal route, yielding a quantum yield of 8.5% [124]. This platform exhibited a linear detection range of 0.01–0.9 µM, a limit of detection of 0.0138 µM, and satisfactory recoveries of 95.6–98.4% in water samples.

Cytokine biomarkers, including C-reactive protein, tumour necrosis factor-α, and interleukin-6, were detected using CDs synthesized from citric acid via microwave-assisted methods [125]. While quantum yield values were not reported, these sensors exhibited linear ranges of 2.50–24.0 pg mL^−1^ for C-reactive protein, 0.25–32.0 pg mL^−1^ for tumour necrosis factor-α, and 1.50–16.0 pg mL^−1^ for interleukin-6, with corresponding limits of detection of 2.50 pg mL^−1^, 0.25 pg mL^−1^, and 1.50 pg mL^−1^, respectively. These platforms demonstrated good analytical accuracy in biologically relevant matrices, including serum, urine, artificial saliva, and sweat, with recoveries ranging from 99.22% to 103.56%. Collectively, these results indicate that CD-based fluorescent biosensors can achieve sensitive and quantitative detection of diverse biological targets. However, their application remains largely confined to biological and food-related matrices, with no validation reported in real municipal wastewater.

Finally, environmental contaminants, such as pesticides and phenolic compounds, are also listed in Table 1, illustrating the extension of CD-based fluorescent sensing platforms to agrochemical and industrially relevant analytes. Pesticide detection was demonstrated for compounds including thiamethoxam, propargite, hexaconazole, and quinalphos using CDs derived from tea waste via hydrothermal synthesis [126]. These CDs exhibited a relatively high quantum yield of 40.05%, indicating strong fluorescence. The reported sensors covered wide linear ranges, including 0.2–5000 ng mL^−1^ for all targets, with limits of detection as low as 0.001 ppm for thiamethoxam and 0.01 ppm for propargite, while the limit of detection for quinalphos reached 0.0002 ppm. Although recovery studies were not reported for these pesticide sensors, the broad dynamic range and low detection limits indicate their suitability for environmental monitoring applications.

Phenolic compound detection is exemplified by tannic acid, monitored using CDs synthesized from xylan via microwave-assisted pyrolysis in the presence of polyethyleneimine [115]. This system exhibited a quantum yield of 8.0%, a linear detection range of 0.1–5 µM, and a limit of detection of 0.04 µM. Validation in lake water and white wine yielded recoveries of 94.0–112.0%, demonstrating good analytical accuracy in complex aqueous and food matrices. Another example is 4-nitrophenol, a toxic organic pollutant commonly associated with industrial effluents, pesticide degradation, and domestic wastewater discharges. The reported fluorescent CD-based sensor for 4-nitrophenol employed waste-derived precursors obtained from *Citri Reticulatae Pericarpium* and was synthesized via a hydrothermal route [116], highlighting the use of green and sustainable carbon sources. Although the quantum yield was not reported, the sensor exhibited a wide linear detection range from 0.2 to 80 µM and achieved a limit of detection of 0.17 µM, within environmentally relevant concentration levels. Validation across multiple aqueous matrices—including tap water, river water, and domestic wastewater—yielded recovery values ranging from 95.45% to 107.38%, demonstrating good analytical accuracy and robustness in complex water samples. Overall, these studies confirm that fluorescent CD-based sensors can be effectively tailored for the detection of diverse environmental contaminants. Nevertheless, as with other target classes, systematic validation in real wastewater matrices remains scarce, underscoring the need for further studies addressing matrix complexity and real-world applicability within wastewater-based epidemiology.

In general, Table 1 highlights the versatility of fluorescent CD-based (bio)sensors for detecting a wide range of wastewater-relevant chemical and biological targets. Across different analyte classes, adequate analytical sensitivity and recovery are generally achieved in complex matrices. However, sensing performance is primarily governed by surface chemistry and recognition strategies rather than fluorescence efficiency alone. A consistent limitation remains the scarce validation in real wastewater matrices. Wastewater is a highly complex and heterogeneous system, containing dissolved organic matter, metal ions, suspended solids, and exhibiting variable pH and ionic strength. These components can significantly affect fluorescence-based detection through multiple mechanisms, including dynamic and static quenching, inner filter effects, and background autofluorescence, leading to signal distortion and reduced sensitivity.

Interactions between matrix components and the surface functional groups of carbon dots may further alter their optical properties, block active binding sites, or induce aggregation under high ionic strength conditions, compromising stability and reproducibility [127,128]. Biofouling and non-specific adsorption also contribute to performance variability, while the intrinsic temporal and spatial variability of wastewater poses additional challenges for method standardization and reproducibility [129]. These factors collectively explain why many CD-based sensing platforms, although highly sensitive under controlled laboratory conditions, still face significant barriers when applied to real wastewater systems [50,130].

In addition to matrix-related challenges, several limitations are inherent to the carbon dot materials themselves. The wide diversity of precursors, synthesis routes, and processing conditions has led to significant variability in CD properties, hindering systematic understanding and limiting the ability to design materials in a target-oriented manner. Although strategies such as heteroatom doping are widely reported to enhance optical performance [131,132], their effects are not always consistent, highlighting the need for a deeper understanding of structure–property relationships [133,134].

Furthermore, bottom-up synthesis approaches may generate fluorescent molecular by-products that can interfere with the intrinsic emission of CDs, potentially leading to misinterpretation of sensing performance [135,136]. The effective removal of these species often relies on purification steps such as dialysis, which are difficult to scale and may increase resource consumption [137]. The lack of standardized protocols to assess purification efficiency further complicates comparisons across studies.

Moreover, the reproducibility of CD synthesis and the lack of harmonized characterization methodologies remain critical barriers to the reliable comparison and practical implementation of these materials [138,139]. In parallel, while waste- and biomass-derived CDs are frequently described as “green”, a more critical evaluation of their environmental sustainability is required. Current assessments are often based primarily on the use of renewable precursors, whereas other key principles of green chemistry—such as atom economy, reaction efficiency, and the generation and toxicity of by-products—are rarely considered. In many cases, synthesis yields remain low, and the environmental impact of residual impurities is not well understood [140,141]. These aspects highlight the need for more rigorous and quantitative sustainability assessments to ensure that CD-based sensing platforms are not only effective but also genuinely aligned with green chemistry principles.

**Table 1:** Overview of fluorescent CD-based (bio) sensors reported in the literature for the detection of chemical and biological targets relevant to human health and wastewater-based epidemiology.

Target and Biorecognition Approach			CD Source and Synthesis Characteristics				Analytical Performance and Validation				Ref.
Target Type	Target	(Bio) Receptor	Natural Precursors or Waste	Other Precursors	Synthesis Method	Quantum Yield (%)	Linear Range (µM)	LOD (µM)	Samples	Recovery (%)	
Metal ions	Fe^3+^	NR	Banana peels	NR	Hydrothermal	2.6–18.06	37–277	6.1	Water	96–100	[101]
	Cu^2+^	NR	Banana juice	NR	Hydrothermal	32	5.5–4400	1.65	Water	High	[102]
	Hg^2+^	NR	NR	Succinic acid and l-Cysteine	Hydrothermal	15	8.05 × 10^13^–4.78 × 10^11^	0.237	Certified material and Primary Drinking Water	101.7–103.7	[103]
	Pb^2+^	NR	NR	Citric acid, glutamine and sodium sulphide	Hydrothermal	10.35	0–25	0.01335	Wastewater and fish species	89.30–116.40 90.22–115.05	[104]
	Fe^3+^	NR	Orange peels	NR	Alkali treatment	7.5	1.0–400.0	0.2	River, irrigation and tap water	99.08–108.24	[105]
	Fe^3+^	NR	Rice husk	NR	Hydrothermal	56.2	0.05–1.15	0.15 0.127	Tablet dosage	99.15–101.6	[106]
Antibiotics	Ciprofloxacin						0–1.3	0.149	tablet supplement	98.53–100.93	
	Ofloxacin									98.56–101	
	Ofloxacin	NR	Sumac	Sumac	Hydrothermal	1.4	0.001–0.09	0.0007	Liquid eye drops and tap water	97–103	[109]
	Tetracycline	NR	spent coffee grounds	NR	Microwave	11.2	0–140	0.36	Water	98.8–105.5	[94]
	Tetracycline	NR	NR	Citric acid	Hydrothermal	18.7	0.0542–0.8	0.0542	Lake water	92.0–106.0	[110]
	Tetracycline	NR	NR	Sucrose and phosphoric acid	Thermostatic water bath	18.8	0–150	0.00173	Swine wastewater	98.3–101.54	[112]
	Kanamycin	Aptamer	NR	Citric acid and formamide	Hydrothermal	39	0.04–0.24	0.018	Milk	89–96.7	[114]
	Amoxicilin	NR	Rice husk	NR	Hydrothermal	55.2	20–1450	101	NR	NR	[108]
	Oxytetracycline	NR	NR	Sucrose	Hydrothermal	NR	2.17–21.7	0.72	Wastewater, milk and meat	NR	[113]
	Oxytetracycline	NR	NR	Citric acid	Hydrothermal	4.8	0–10	0.0096	River and tap water	95.0–109.2	[111]
Inorganic ions	F^−^	NR	NR	Citric acid, glutamine and sodium sulphide	Hydrothermal	10.35	0–12	0.04317	Fish extracts and wastewater	89.30–116.40 90.22–115.05	[104]
	CN^−^	NR	Corn silks	NR	Hydrothermal	15	0.5–50	0.459	Industrial effluent, Sorghum leaves, Flaxseeds	NR	[107]
Bacteria	*E. coli*	Aptamer	Orange peel	NR	Microwave	16.2	500–10^6^ CFU mL^−1^	487 CFU mL^−1^	Milk	NR	[118]
	*E. coli*	Aptamer	Carrot juice	NR	Hydrothermal	NR	1 × 10^2^–1 × 10^8^ CFU mL^−1^	60 CFU mL^−1^	NR	NR	[119]
	*E. coli*	Oligonucleotide	NR	Citric acid	Hydrothermal	NR	0.00001–0.1	0.00103	Chicken, meat and cheese	NR	[120]
	*H. pylori*	ssDNA	NR	Citric acid; malic acid	Microwave	23.0	1.30–11.49	0.098	Saliva	93.06–101.85	[121]
Virus	SARS-CoV-2	Antibody	NR	Thiourea and Citric acid	Hydrothermal	NR	100 μg mL^−1^–100 pg mL^−1^	100 ng mL^−1^	NR	NR	[122]
	SARS-CoV-2	Antibody	NR	purchased		71.83	0.1–100 ng mL^−1^	0.05 ng mL^−1^	Serum	110.05–115.45	[123]
Hormones	Progesterone	Antibody	NR	Citric acid	Hydrothermal	8.5	0.01–0.9	0.0138	water	95.6–98.4	[124]
Cytokine biomarkers	C-reactive protein	NR	NR	Citric acid	Microwave	NR	2.50–24.0 pg mL^−1^	2.50 pg mL^−1^	Serum, urine, artificial saliva and sweat	99.69–103.58	[125]
	Tumour necrosis factor-alpha						0.25–3.20 pg mL^−1^	0.25 pg mL^−1^		99.23–102.98	
	Interleukin-6						1.50–16.0 pg mL^−1^	1.50 pg mL^−1^		99.22–103.56	
Pesticides	thiamethoxam 25 WG	NR	Tea		Hydrothermal	40.05	0.2–5000 ng mL^−1^	0.001 ppm	NR	NR	[126]
	propargite 57 EC	NR		NR				0.01 ppm			
	hexaconazole 5 EC	NR		NR				NA			
	quinalphos 25 EC	NR		NR				0.0002 ppm			
Phenolic compounds	Tannic acid	NR	Xylan	Polyethyleneimine	Microwave assisted pyrolysis	8.0	0.1–5	0.04	Lake water and white wine	94.0–112.0	[115]
	4-Nitrophenol	NR	*Citri Reticulatae Pericarpium*	NR	Hydrothermal	NR	0.2–80	0.17	Tap water, domestic wastewater and river water	95.45–107.38	[116]
Biothiols	Cysteamine, dithiothreitol, mercaptosuccinic acid, glutathione, mercaptoacetic acid, and mercaptoethano	NR	NR	Glycine, histidine and leucine	Microwave	10.32–17.19	100–600	30	NR	NR	[142]
Flavonoids	Morin	NR	*Acorus calamus* rhizome	NR	Hydrothermal	15	0–2	0.096	Human urine	98.7–102.2	[117]

NR—Not reported; For biological targets, limits of detection are reported in the original biological units (e.g., CFU mL^−1^ or pg mL^−1^), as direct molar conversion is not applicable.

## 4. Conclusions

This review highlights the growing potential of carbon dot-based fluorescent (bio)sensors as alternative analytical tools for wastewater-based epidemiology. Compared to conventional techniques, these platforms offer advantages in terms of cost, portability, rapid response, and compatibility with complex aqueous matrices, while enabling the detection of a broad range of chemical and biological targets.

Despite these advances, the field remains largely at the proof-of-concept stage, with limited validation under real wastewater conditions. The complexity and variability of wastewater matrices, combined with challenges related to reproducibility and standardization, continue to hinder the translation of these sensing platforms into practical applications. Bridging this gap will require not only improved analytical performance but also systematic validation strategies and the development of more robust and reliable sensing systems capable of operating under realistic conditions.

From a broader perspective, advancing this field requires addressing fundamental challenges associated with carbon dot materials, including the lack of reproducible and target-oriented synthesis, the presence of fluorescent by-products that complicate signal interpretation, and the absence of scalable and standardized purification protocols. In parallel, the environmental sustainability of waste-derived CDs remains insufficiently assessed, particularly regarding synthesis efficiency and the impact of residual impurities. This reflects a broader transition in the field—from sensitivity-driven optimization toward application-oriented design—where robustness, reproducibility, and real-world performance become central. Addressing these challenges will be essential for translating CD-based sensing platforms into reliable and deployable tools for environmental and public health monitoring.

## Data Availability

No data was used for the research described in the article.
